# A Framework for Trajectory Prediction of Preceding Target Vehicles in Urban Scenario Using Multi-Sensor Fusion

**DOI:** 10.3390/s22134808

**Published:** 2022-06-25

**Authors:** Bin Zou, Wenbo Li, Xianjun Hou, Luqi Tang, Quan Yuan

**Affiliations:** 1 Hubei Key Laboratory of Advanced Technology for Automotive Components, Wuhan University of Technology, Wuhan 430070, China; zoubin@whut.edu.cn (B.Z.); sea_rivers@sina.com (W.L.); tlq20080804@whut.edu.cn (L.T.); 231943@whut.edu.cn (Q.Y.); 2 Hubei Collaborative Innovation Center for Automotive Components Technology, Wuhan University of Technology, Wuhan 430070, China; 3 Hubei Research Center for New Energy and Intelligent Connected Vehicle, Wuhan 430070, China

**Keywords:** trajectory prediction, transformer, cluster, multi-sensor fusion, detection and tracking, different driving direction

## Abstract

Preceding vehicles have a significant impact on the safety of the vehicle, whether or not it has the same driving direction as an ego-vehicle. Reliable trajectory prediction of preceding vehicles is crucial for making safer planning. In this paper, we propose a framework for trajectory prediction of preceding target vehicles in an urban scenario using multi-sensor fusion. First, the preceding target vehicles historical trajectory is acquired using LIDAR, camera, and combined inertial navigation system fusion in the dynamic scene. Next, the Savitzky–Golay filter is taken to smooth the vehicle trajectory. Then, two transformer-based networks are built to predict preceding target vehicles’ future trajectory, which are the traditional transformer and the cluster-based transformer. In a traditional transformer, preceding target vehicles trajectories are predicted using velocities in the X-axis and Y-axis. In the cluster-based transformer, the k-means algorithm and transformer are combined to predict trajectory in a high-dimensional space based on classification. Driving data from the real-world environment in Wuhan, China, are collected to train and validate the proposed preceding target vehicles trajectory prediction algorithm in the experiments. The result of the performance analysis confirms that the proposed two transformers methods can effectively predict the trajectory using multi-sensor fusion and cluster-based transformer method can achieve better performance than the traditional transformer.

## 1. Introduction

Automated vehicles and advanced driver assistance systems (ADAS) have received a surge of attention in recent years as they are considered to be an effective solution for traffic congestion and safety [1,2,3]. Reliable trajectory prediction of preceding vehicles is crucial for the planning and decision-making of automated vehicles. Compared to studies of surrounding vehicles trajectory prediction [4], preceding target vehicles (PTVs) should receive more attention, which in turn has a higher possibility of risk to the automated ego-vehicle (EV). Based on the future trajectory of PTVs, the EV can generate a more comfortable and safe path, avoiding or mitigating the risk of collision [5].

**Figure 1:**
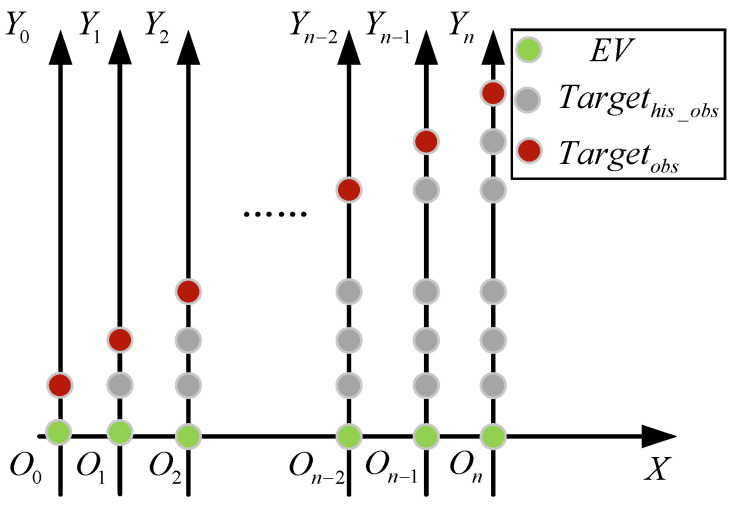
Relative motion of the PTV.

A major reason for the prosperity of vehicle trajectory prediction algorithms is the availability of public datasets [6,7], which assists researchers in quickly validating their algorithms. Despite these favorable results on public datasets, when the vehicle trajectory prediction system evaluated, not only should the accuracy of the prediction should be considered, but also the generalization ability of the established model should be evaluated; that is, whether the model can accurately predict the trajectory of the vehicle in real road driving. Vehicle-to-vehicle (V2V) is also a source for trajectory prediction input. However, in the case that the V2V communication technique is unavailable, autonomous vehicles cannot receive accurate information from surrounding vehicles, and the autonomous vehicles have to deduce the trajectory of other vehicles through various onboard sensors [8,9,10]. Currently, a lot of research has captured PTVs using cameras, LIDAR, and other sensors in real road driving [10,11,12]. However, the sensor is moving because it is fixed to EV, which results in PTV positions that are not in the same coordinate system. Figure 1 shows the results of the PTV in a moving EV vehicle coordinate system and a stationary EV vehicle coordinate system, respectively, where the green points represent the EV position, the gray points represent observed PTV history position, and the red points represent real PTV position. When EV and PTV are driving at the same speed in the X-axis, PTV is traveling in the Y-axis as observed by the EV sensor. For this reason, when predicting vehicle trajectories in real roads, it is necessary to focus not only on excellent prediction algorithms to predict future trajectories but also on methods to obtain historical trajectories of PTVs. The goal of this paper is to develop such a system.

In this paper, we propose a PTV trajectory prediction system based on two different transformer methods using LIDAR, camera, and combined inertial navigation system fusion. First, the relative position of PTV and EV is obtained by LIDAR–camera fusion. This process consists of vehicle detection, tracking using image, and relative position detection of lateral and longitudinal positions using LIDAR. Second, a PTV trajectory extraction method is developed, which converts the PTV in the dynamic EV vehicle coordinate system to a world coordinates system in order to generate historical trajectories. Third, a Savitzky–Golay (S-G) filter is employed for moving to smooth trajectory curves. Next, two transformer-based methods are built to predict the PTV’s future trajectory. Finally, the models are trained and validated using the raw longitude and latitude, image and point cloud datasets collected at the real urban road in Wuhan, China. Our main contributions are summarized as follows:

(1) Most trajectory prediction algorithms are validated by public datasets that provide the location directly. Therefore, the whole process of vehicle trajectory prediction cannot be systematically considered. We come up with a framework for PTV’s trajectory prediction using the real driving process collected from LIDAR, camera, and combined inertial navigation system fusion.
(2) Vehicle trajectory prediction algorithms based on LSTM and its variants are difficult to model due to complex temporal dependencies. Therefore, the other contribution is two different transformer-based methods built to the PTV’s future trajectory.

The remainder of this paper is organized as follows. Section 2 introduces the pre-processing of the raw point cloud from 3D LIDAR, image, and longitude and latitude from EV and generating PTV’s historical trajectory. Section 3 presents two transformer-based PTV trajectory prediction models. Section 4 describes the process of dataset creating, model training and results analysis. Finally, Section 5 offers the conclusions and future work.

## 2. Related Work

Vehicle driving is a continuous, time-varying, and dynamic process. Extensive research has been conducted on vehicle trajectory prediction. For the PTVs’ trajectories prediction, prior works can be divided into two categories: model-driven methods and data-driven methods [13].

The model-driven methods include the hidden Markov model (HMM), Gaussian mixture model (GMM), vehicle dynamics model (VDM), and polynomial model (PM). Ye et al. [14] proposed a novel vehicle trajectory prediction algorithm named double hidden Markov of trajectory prediction (DHMTP). The algorithm was based on a hidden Markov model with double hidden states and predicted the vehicle trajectory at multiple subsequent moments. Wiest et al. [15] built a mixture Gaussian–Bayesian model-based variational probabilistic trajectory, in which Gaussian and Bayesian methods were jointly utilized to predict future vehicle coordinates. The validation of real word trajectory showed that the proposed model could predict future vehicle trajectories within two seconds. VDM represented a model built using vehicle dynamics data (e.g., velocity, acceleration, steering angle, and yaw angle) and relevant mathematical methods. Vehicle kinematic data and a maneuver identification model were combined to trajectory prediction model which was validated by real driving data [16]. The results indicated that the model was effective in short-term prediction. However, the accuracy of its long-term prediction was not stable. PM was usually employed to fit non-linear curves. Guo et al. [17] fitted and predicted the longitudinal trajectory of a vehicle using a fifth-order polynomial. These approaches only achieved favorable results on short-term trajectory forecasts. However, it did not show promising results when predicting long trajectories.

Besides the traditional methods motioned above, a large number of works focused on vehicle trajectory prediction by using recurrent neural network (RNN) and Long short-term memory (LSTM). Especially variant LSTM had received a lot of attention from researchers. Deo and Trivedi [18] used the LSTM encoder to encode the trajectory vectors of surrounding vehicles to predict the future trajectory and validated the effect on the NGSIM dataset. In [19], two streams graph LSTM to predict trajectories and driving behavior were adopted under urban scenarios. The first stream used only a conventional LSTM encoder-decoder network when the second stream used a weighted dynamic geometric graph. The model was evaluated on the Argoverse, Lyft, Apolloscape, and NGSIM datasets. Although it had achieved promising results in long-term trajectory prediction, LSTM normally had difficulty modeling complex temporal dependencies [20].

Recently, Transformer networks had made ground-breaking progress in Natural Language Processing domains (NLP) [21]. Transformers discarded the sequence of language sequences and only modeled temporal dependencies using a powerful self-attention mechanism. The key advantage of the transformer architecture was the significant improvement in temporal modeling compared to RNN. Several studies had used transformer networks to model pedestrian trajectory prediction and achieved good results [22,23,24]. Although the transformer was excellent at predicting pedestrian trajectories, vehicles had faster speeds compared to pedestrians. Moreover, these studies had not been able to predict target trajectory from the raw data because the location of the targets was already provided in the public dataset. In this work, we generate history trajectories using raw data from sensors and predict PTV’s future trajectory based on two different transformer methods.

## 3. Sensors Fusion and History Trajectory Generation

### 3.1. Detection

Detection and tracking are prerequisites for generating PTV’s historical trajectory. Moreover, excellent trackers depend largely on a superb detector. You only look once (YOLO) algorithms can achieve faster performance than the two-stage algorithm by tuning the backbone network due to the omission of the coarse localization process. In particular, the YOLOv5 model is faster, more accurate and has a lower number of model parameters than the YOLOv4 model [25]. Therefore, YOLOv5 is employed as the detector for PTV.

### 3.2. Tracking

DeepSORT is an improved version of simple online and real-time tracking (SORT). It integrates a pre-trained neural network to generate feature vectors which are used as a deep association metric. Specifically, it applies a trained convolutional neural network (CNN) to detect obstacles on large-scale datasets. By using this network integration, deepSORT overcomes the shortcomings of SORT while ensuring that the system is easy to implement, effective, and suitable for real-time situations [26]. Hence, we apply deepSORT as the tracker.

### 3.3. Lidar-Camera Fusion

Sensor fusion can enhance sensing capabilities and reduce costs by exploiting the complementary properties. LIDAR provides accurate PTV geometry information; however, the LIDAR has low resolution and a low frame rate. On the contrary, monocular cameras have high frame rates and resolution but difficulty in perceiving 3D geometric information. Therefore, camera–LIDAR fusion has been more focused on the perception of autonomous driving [27].

**Figure 2:**
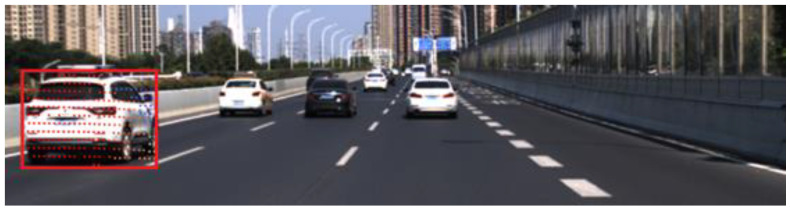
The effect of LIDAR–camera fusion.

The process of LIDAR-camera fusion is as follows. First, the 3D point cloud is cropped according to EV’s driving direction. Next, the YOLOv5 detector fetches the PTV’s bounding box from the image. Then, point clouds are projected and clustered in the pixel coordinate system according to the joint calibration parameters of the LIDAR and the camera. After that, the position of PTV relative to EV is extracted based on the clustered point cloud. Finally, temporal features of the PTV are associated with the deepSORT tracker. Figure 2 displays the effect of LIDAR–camera fusion.

### 3.4. History Trajectory Generation

**Figure 3:**
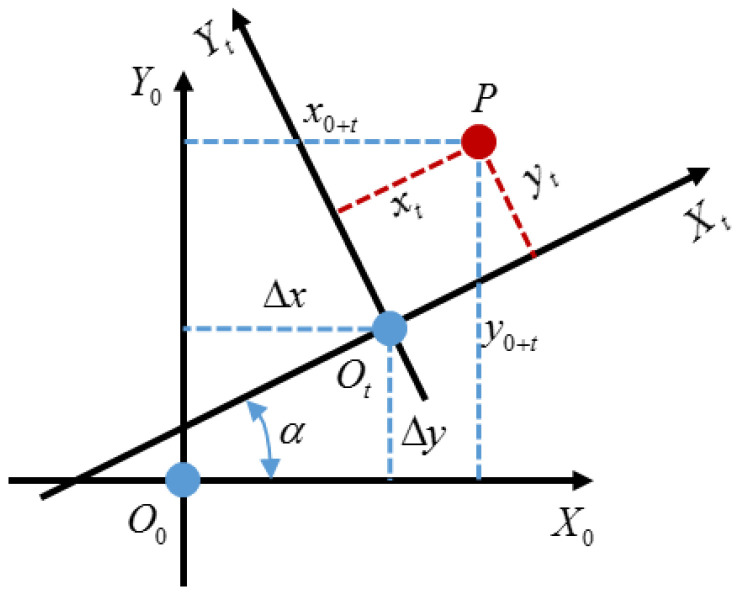
EV vehicle coordinate systems unification method.

Predicting the PTV’s trajectory requires the position in continuous time and the same coordinate system. However, the PTV’s position is captured on a moving EV vehicle coordinate system and PTV coordinates are constantly changing. It means the PTV positions observed by the sensors are not in the same coordinate system. Therefore, it is necessary to transform the EV coordinate systems at a different time into a unified the EV vehicle coordinate system. Figure 3 shows the EV vehicle coordinate systems unification method.

**Figure 4:**
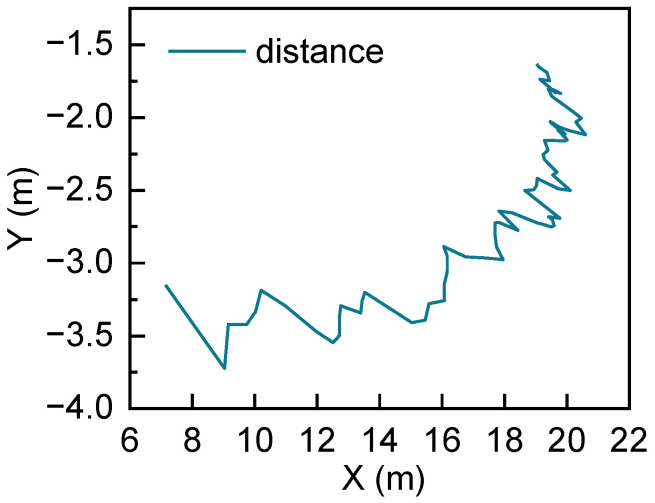
Relative distance of PTV and EV.

Here, we take an example that a trajectory starting point and time 0 of the world coordinates $X_{0}O_{0}Y_{0}$, and convert the PTV’s position at time *t* of the world coordinates $X_{t}O_{t}Y_{t}$ into $X_{0}O_{0}Y_{0}$ as shown in Figure 3. Where $O_{0}$ and $O_{t}$ are the position of EV at time 0 and time t, respectively. P represents the position of PTV relative to EV at time t. $\left(x_{t},y_{t}\right)$ is the coordinates of the PTV in the EV coordinate system at time t. $\left(x_{0+t},y_{0+t}\right)$ is PTV’s position at time t under the EV coordinate system at time 0. Δx and Δy are Y-axis and X-axis displacement from EV at time 0 and time t which are calculated from combined inertial navigation system output, respectively. Besides, α represents EV’s heading angle deviation at time 0 and time t. According to geometry, $\left(x_{t},y_{t}\right)$ is converted to $\left(x_{0+t},y_{0+t}\right)$ depending on (1). Additionally, it is translated into matrix form as (2). The relative distances between the X-axis and Y-axis of the PTV and EV are shown in Figure 4.

(1) $$\begin{array}{l} x_{0+t}=\Delta x+x_{t}\cos\alpha -y_{t}\sin\alpha \\ y_{0+t}=\Delta y+x_{t}\sin\alpha +y_{t}\cos\alpha \end{array}$$

(2) $$[\begin{array}{c} x_{0+t}\\ y_{0+t} \end{array}]= [\begin{array}{cc} \cos\alpha & -\sin\alpha \\ \sin\alpha & \cos\alpha \end{array}] [\begin{array}{c} x_{t}\\ y_{t} \end{array}]+ [\begin{array}{c} \Delta x\\ \Delta y \end{array}]$$

**Figure 5:**
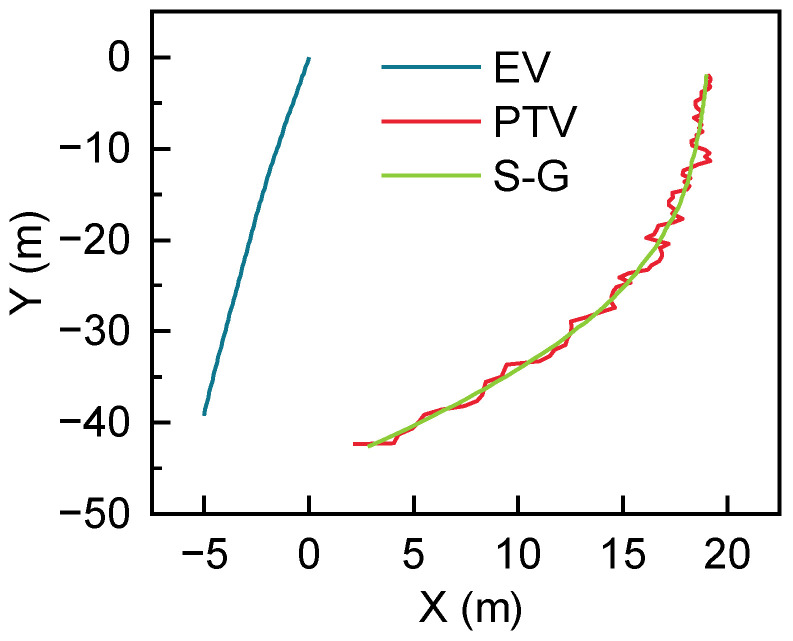
Historical trajectory generation.

Once the coordinates of the EV have been unified at the same time, it is necessary to convert all trajectories into the world coordinates using the latitude, longitude and time information provided by the EV’s combined inertial navigation system. The converted PTV trajectories are smoothed by the S-G filter [28]. Figure 5 shows the EV trajectory in blue, the PTV tracking trajectory in red and the filtered PTV trajectory in green. As can be seen from Figure 5, the filtered data are smooth sufficient to be used in a vehicle trajectory prediction model.

## 4. Proposed Model

In urban scenes, there is wide variation in speed and direction of PTV under the world coordinate system, especially opposite lanes and intersections. In this case, the traditional Transformer (TF) and cluster-based Transformer (C-TF) models are developed, respectively, to predict PTV’s future trajectory.

### 4.1. Transformer Network

There are two modules (the encoding block and the decoding block) in the transformer network. The encoding block mainly consists of multi-head attention, add & norm, and feed-forward. The decoding block is mainly made up of masked multi-head-attention, multi-head attention, add & norm, and feed-forward. The transformer network captures the dependencies of time-series data and the non-linear features of spatial data primarily through the attention mechanism, which includes the self-attention mechanism and multi-head attention mechanism [23].

#### 4.1.1. Self-Attention Mechanism

Self-attention takes query matrix *Q*, key matrix *K*, value matrix *V*, dimension of queries and keys as input, which is calculated by a dot product of *Q* and *K*, and scaled factor of $1/d_{k}$. Then, a softmax function is employed to obtain the weights of V. Self-attention mechanism is formulated as in (3).

(3) $$Q_{i}=QW_{i}^{Q},K_{i}=KW_{i}^{K},V_{i}=VW_{i}^{V}\;Attention(Q_{i},K_{i},V_{i})=soft\max(QK^{T}/\sqrt{d_{k}}),i=1,\ldots ,n$$

#### 4.1.2. Multi-Head Attention Mechanism

Instead of learning a single attention function, [21] has found that it is more profitable to map the queries, keys, and values for h times to learn different contextual information, respectively. In this case, the self-attention function is run in parallel on each projected version of queries, keys, and values. Next, the results are concatenated and projected again to produce the weight of final values. Therefore, the transformer structure can jointly produce a full-scale latent feature of trajectory data from different representation subspaces based on the multi-head attention. The multi-head attention mechanism is calculated by (4).

(4) $$head_{i}=Attention(Q_{i},K_{i},V_{i}),\;i=1,\ldots ,n\;MultiHead(Q,K,V)=Conc(head_{1},\ldots ,head_{n}{)W}^{o}$$

#### 4.1.3. Feed-Forward Networks

The fully connected feed-forward networks are composed of two linear transformations with a ReLU activation and applied to each attention sub-layers. In addition, the residual dropout module is set to improve the transformer network efficiency. Feed-forward networks are given by Equation (5).

(5) $$Feedforward(x)=\max(0,{xW}_{1}+b_{1})W_{2}+b_{2}$$

#### 4.1.4. Positional Encoding

The transformer does not include any processing of the sequences, so it requires positional encoding to allow time series features to be expressed in the network. In other words, with the positional encoding, each input embedding is assigned a time feature. [21] employed trigonometric functions to construct position vectors which have equal dimension for different positions. The formula is as follows:

(6) $$p_{i}^{\left(t\right)}=\left\{\begin{array}{c} \sin(\frac{t}{10000^{i/d}}),\;\mathrm{for}\;i\;\mathrm{even}\\ \cos(\frac{t}{10000^{i/d}}),\;\mathrm{for}\;i\;\mathrm{odd} \end{array}\right.$$

where d is mapping dimensions, i represents positions where PTV’s velocity value appears.

### 4.2. TF Model

**Figure 6:**
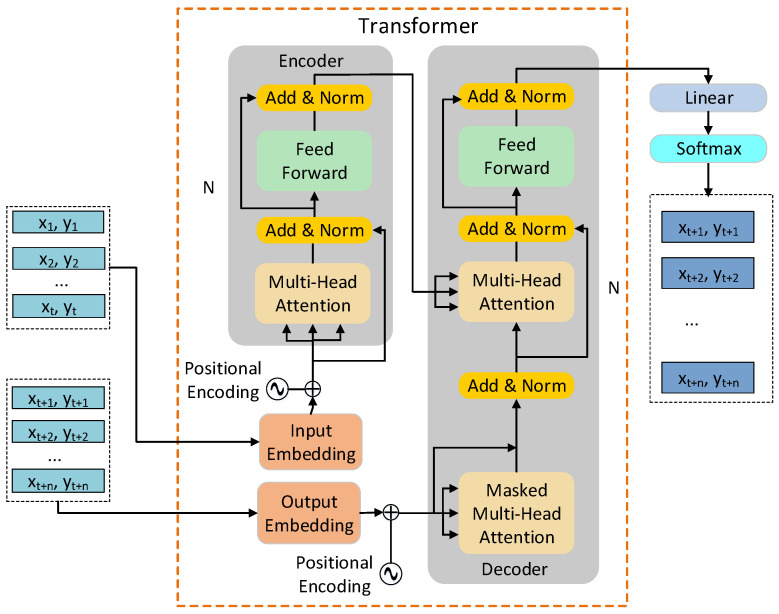
The TF network.

The transformer network predicts PTV’s future trajectories by processing history trajectories and current positions. For PTV observation points P, we define the historical trajectory $P_{his}=(p^{\left(1\right)},p^{\left(2\right)},\ldots ,p^{\left(t\right)})$, predicted trajectory $P_{his}=(p^{(t+1)},p^{(t+2)},\ldots ,p^{\left(t+n\right)})$, where t is observation time step and n is the prediction time step. $p^{\left(t\right)}$ is represented by $p^{\left(t\right)}=(v_{x}^{t},v_{x}^{t})$. When new trajectory features $P_{his}=(p^{\left(1\right)},p^{\left(2\right)},\ldots ,p^{\left(t\right)})$ are fed into the transformer network, input embedding maps the velocity $p^{\left(t\right)}$ to a higher space d at time t before positional encoding. Next, position encoding is employed to supplement the timing characteristics. The feature vectors are transferred to the encoder and successively pass through two residual networks to extract features, which are the multi-head attention mechanism residual network and the feed-forward neural network residual network, respectively. Each residual network is followed by layer normalization. The encoding process is repeated six times. The future trajectory $P_{pred}^{C}=\{k_{i}|i=t+1,t+2,\ldots ,t+n\}$ will also be mapped with output embedding and positional encoding, but it is immediately followed by the masked multi-headed attention mechanism residual network and layer normalization. These feature vectors are then fused with the output vectors of the encoder. Then, the features are further extracted by the same head attention mechanism residual network and feed-forward neural network residual network. The decoding process is also repeated six times. Finally, after the linear and softmax layers, the final result is output. The TF network is presented in Figure 6.

### 4.3. C-TF Model

**Figure 7:**
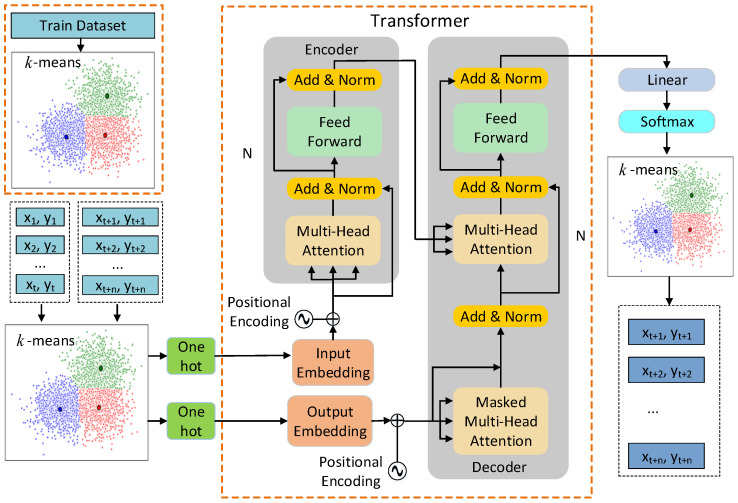
The C-TF network.

Transformer network architecture for C-TF is the same as TF. However, there is a great difference in what is trained. Firstly, the k-means clustering algorithm clusters the trajectory features in the training dataset. Then, when new trajectory features $P_{his}=(p^{\left(1\right)},p^{\left(2\right)},\ldots ,p^{\left(t\right)})$ are fed into the equation, the k-means algorithm matches $p^{\left(t\right)}$ to the centroid each cluster of train dataset. In this way, $p^{\left(t\right)}$ is converted to cluster space $P^{C}=\{k_{i}|i=1,2,\ldots ,t+n\}$. In the transformer network, the historical trajectories $P_{his}^{C}=\{k_{i}|i=1,2,\ldots ,t\}$ in the cluster space are mapped again by the input embedding, then position encoding is employed to supplement the timing characteristics. The feature vectors in the cluster space are transferred to the encoder and successively pass through two residual networks to extract features, which are the multi-head attention mechanism residual network and the feed-forward neural network residual network, respectively. Each residual network is followed by layer normalization. The encoding process is repeated six times. The future trajectory $P_{pred}^{C}=\{k_{i}|i=t+1,t+2,\ldots ,t+n\}$ will also be mapped with output embedding and positional encoding, but will be immediately followed by the masked multi-headed attention mechanism residual network and layer normalization. These feature vectors are then fused with the output vectors of the encoder. Next, the features are further extracted by the same head attention mechanism residual network and feed-forward neural network residual network. The decoding process is also repeated six times. The feature vectors output by the decoder are mapped into temporal cluster space features after the linear and softmax layers. The cluster space features are matched with the clusters of train dataset to obtain the corresponding centroid. The final predicted trajectory is represented by the centroid of the time series. The C-TF network is shown in Figure 7.

k-means is an algorithm that regards distance as the feature, which considers clusters to be composed of objects that are close in distance. Some research has employed k-means in vehicle trajectory clustering [29]. k-means clustering identifies which cluster is belonged to every element in the dataset with the nearest mean. The number of means, k to be generated is equal to the number of clusters, k. It runs iteratively by finding the minimum squared error between the means, c of the cluster and the observed data. In each round of iteration, there is a new set of means generated and it stops when the convergence of squared error minimization in k-means as (7).

(7) $$min\sum_{i=1}^{N}\sum_{j=1}^{k}{\left\|o_{i}-c_{j}\right\|}^{2}$$

where N denotes the number of observations and o represents observed data.

## 5. Experiment and Result Analysis

In this section, the trajectory prediction methods are evaluated in real driving data compared with LSTM using naturalistic driving data under the urban scenario.

### 5.1. Driving Data Collection

**Figure 8:**
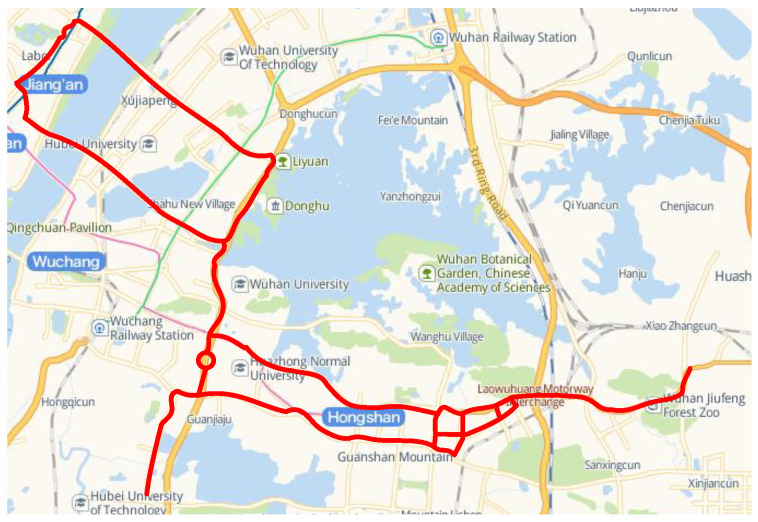
Data collection routes.

**Figure 9:**
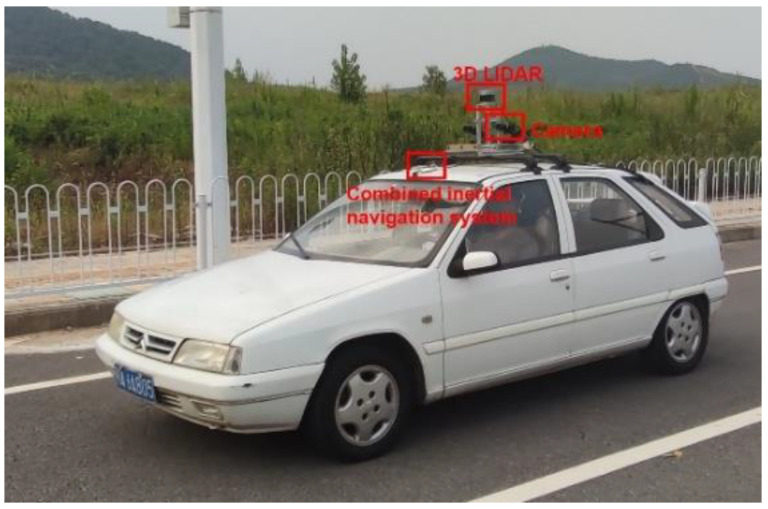
Test vehicle.

We have collected a large amount of vehicle trajectory data on an urban scenario in Wuhan, China. Routes are shown in Figure 8. We have collected over 90 km of naturalistic driving data including as many road conditions as possible, at different times of the day. The test vehicle (see Figure 9) is equipped with OXTS RT3002 combined inertial navigation system, Velodyne VLP-32C LIDAR, and FLIR Grasshopper3 GS3-U3-23S6C camera. All sensors are time-synchronized at 10 Hz. In addition, the camera is calibrated with an intrinsic matrix and then all sensors are calibrated with an extrinsic matrix to space-synchronization. All data are collected based on the ROS Melodic version of the operating system under the Ubuntu 18.04 system. Same length settings as Argoverse’s Motion Forecasting dataset [6], we selected PTVs that are recorded for longer than 5 s, and the trajectories are cropped to 5 s per set that the first 2 s are as input and the last 3 s are prediction. There are 727 PTVs, with a total of 58,185 sets of trajectory data extracted. The data are divided into a training set, a validation set, and a test set using 6:2:2.

**Figure 10:**
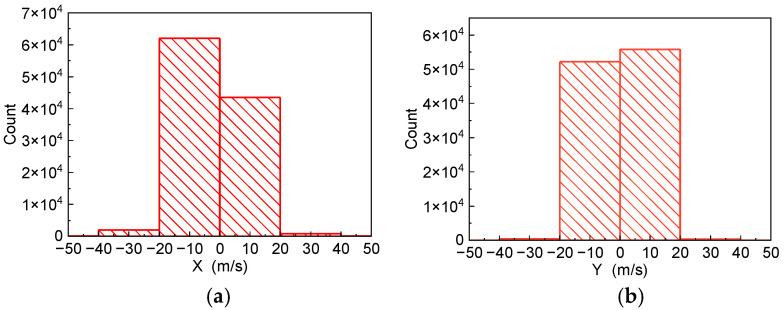
Velocity distribution. (**a**) Velocity distribution of X-axis. (**b**) Velocity distribution of Y-axis.

As the algorithms in this paper are trained by velocity, we present the PTV velocity distribution in the X-axis and Y-axis as Figure 10 where Figure 10a,b show the velocity distribution in the X-axis and Y-axis, respectively. It can be noticed from Figure 10 that the speed data are with a uniform distribution of direction and value.

### 5.2. Implementation Details

We perform all our experiments in PyTorch with CUDA 10.2 and the cuDNN backend. All experiments run on two NVIDIA GTX-1080Ti GPUs and have a maximum batch size of 200. For TF, we set six layers and eight attention heads. In addition, we adopt an L2-loss function and Adam optimizer. Besides this, we normalize the PTV’s velocity in the train dataset. For C-TF, we cluster the velocities in the X-axis and Y-axis directions in 6000 clusters. We also work with six layers and eight attention heads and the Adam optimizer. What we need to highlight is the cross-entropy loss function that is employed in C-TF. The trajectories are 5 s per set, in which the input data are 20 points, and the output data are 30 points since the sampling frequency is 10 Hz.

### 5.3. Evaluaiton Metrics

In this paper, the average displacement error (ADE) and final displacement error (FDE), are employed to evaluate the vehicle forecast prediction error.

ADE represents the average difference of the Euclidean distance between each prediction position and the true trajectory, which is given by (8).

(8) $$\mathrm{ADE}=\frac{1}{T}\sum_{t=1}^{T}\sqrt{{\left(x_{i}^{pred}-x_{i}^{gt}\right)}^{2}+{\left(y_{i}^{pred}-y_{i}^{gt}\right)}^{2}}$$

FDE denotes the Euclidean distance difference on end position between the predicted trajectory and the true trajectory, which is given by (9).

(9) $$\mathrm{FDE}=\sqrt{{\left(x_{T}^{pred}-x_{T}^{gt}\right)}^{2}+{\left(y_{T}^{pred}-y_{T}^{gt}\right)}^{2}}$$

where T represents predicted length, $x_{i}^{pred},y_{i}^{pred}$ denote the predicted position at the time i and $x_{i}^{gt},y_{i}^{gt}$ represent the true position at the ith moment.

### 5.4. Result Analysis

Table 1 shows the ADE and FDE for the LSTM, TF, and C-TF models at 1, 2, and 3 s. It is apparent from this table that the proposed methods TF and C-TF have achieved a large improvement compared to LSTM both ADE and FDE at 1, 2, and 3 s. TF improves ADE by 3.202 m, 6.780 m, 8.271 m compared to LSTM at 1, 2, and 3 s and improves FDE by 6.595 m, 13.318 m, 18.254 m, respectively. In addition, C-TF receives better results compared to TF. For ADE, C-TF shows an improvement of 3.507 m, 7.392 m, and 10.460 m at 1, 2, and 3 s, respectively, compared to LSTM. In addition, there are improvements of 7.034 m, 14.716 m, 20.781 m for FDE at 1, 2, and 3 s, respectively.

**Figure 11:**
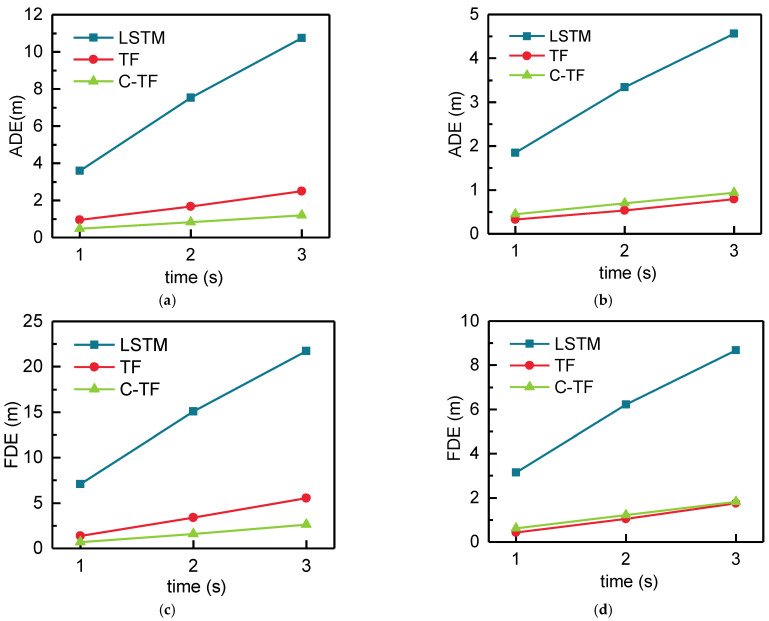
The ADE and FDE in the X- and Y-axis directions. (**a**) The ADE of the X-axis. (**b**) The ADE of the Y-axis. (**c**) The FDE of the X-axis. (**d**) The FDE of the Y-axis.

Figure 11a–d presents the ADE and FDE in the X-axis and Y-axis directions respectively. Both the proposed TF and C-TF achieve a large improvement over the LSTM as can be seen from Figure 11. For ADE and FDE in the x-axis direction, C-TF has better results than TF. For ADE, C-TF improves by 0.466 m, 0.857 m, and 1.298 m compared to TF and FDE improves by 0.688 m, 1.775 m, and 2.942 m at 1, 2, and 3 s, respectively. However, in the Y-axis direction, TF has a better effect compared to C-TF for both ADE and FDE. There is an improvement of 0.122 m, 0.161 m, and 0.151 m for ADE, TF compared to C-TF, 0.191 m, 0.180 m, 0.070 m for FDE. Although the TF has better results than the C-TF in the Y-direction, the improvement over the X-axis C-TF is slightly less apparent.

**Figure 12:**
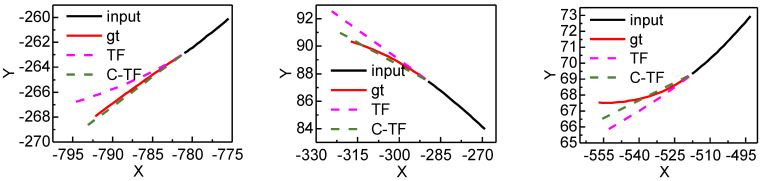
PTV The predicted effect of C-TF and TF on real-world preceding vehicles dataset.

Figure 12 shows the predicted trajectories of C-TF and TF for different positions and directions. The black solid line is the historical trajectory, the red solid line is the ground truth, the pink dashed line is the TF prediction result, and the green dashed line is the C-TF prediction result.

**Figure 13:**
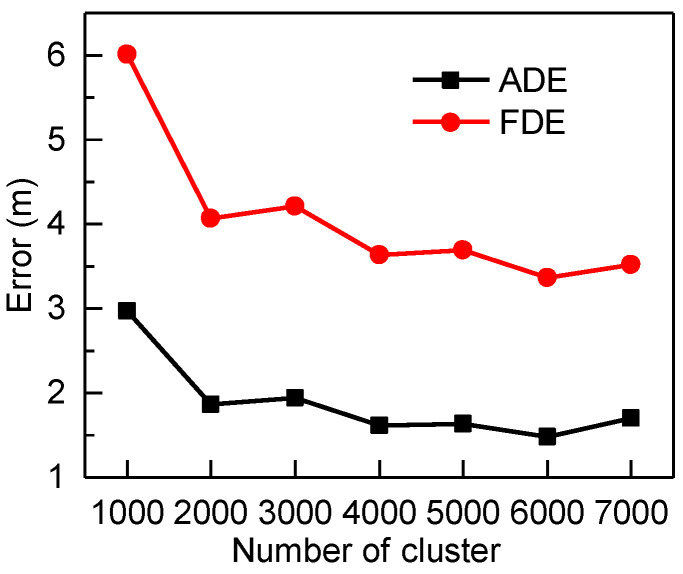
The 3rd second accuracy of C-TF at different number of clusters.

As shown in Table 1 and Figure 11, the C-TF model has the best prediction. However, the effect of the C-TF model is influenced by the number of clusters. Figure 13 shows the ADE and FDE of C-TF at the 3rd second when the training dataset is clustered by k-means into different numbers of clusters. As can be seen from Figure 13, the predictive effect of C-TF gradually improves as the number of clusters increases. C-TF has the best prediction when the training dataset is clustered in 6000 clusters. There is a small accuracy decrease of C-TF at 7000 clusters. However, when observing the overall trend, it can be drawn that the model’s predictions stabilize when the number of clusters reaches 6000. One interesting finding is TF accepts higher prediction accuracy than C-TF when clusters are below 1000. However, when the number of clusters exceeds 1000, C-TF obtains better results. A possible explanation for this might be that k-means clustering algorithm fuses X and Y direction trajectory parameters into clusters. As more clusters are clustered, each set of data represented by a cluster becomes closer to the centroid. As a result, the centroid of the clusters matched by k-means after the softmax layer is closer to the true value in the data parsing module.

**Figure 14:**
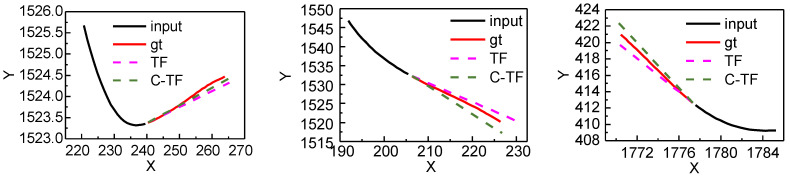
PTV The predicted effect of C-TF and TF on the Argoverse dataset.

A further validation was done on the Argoverse dataset in order to verify the generalizability of the proposed model. Argoverse dataset is a motion prediction benchmark that has collected more than 30 K scenes in Pittsburgh and Miami. Each scene is a sequence of frames sampled at 10 HZ. Each sequence has an object called an “agent”, and the task of trajectory prediction is to predict the position of the agent in the future range of 3 s. These sequences are divided into training, validation, and test sets with 205,942, 39,472, and 78,143 sequences, respectively. These splits do not overlap geographically. For the training and validation sets, each sequence lasts 5 s. The first two seconds are used as input data, and the other 3 s are used as the underlying facts of the model’s predictions. For test sets. Only the first 2 s of data are provided. Each frame is given in the form of the coordinates of the center point of all objects in the scene. The center coordinates of all objects in the scene. Here, the training and validation datasets are integrated and split back into three parts: training, validation and testing, according to the ratio of 6:2:2. The first 2 s are taken as history trajectory and the last 3 s are taken as predicted trajectory. The results are presented in Table 2. As can be seen in Table 2, the proposed methods TF and C-TF have also achieved a large improvement compared to LSTM both ADE and FDE at 1,2,3 s. C-TF also has better prediction accuracy than TF. Figure 14 shows the predicted trajectories of C-TF and TF for different positions and directions on the Argoverse dataset. The black solid line is the historical trajectory, the red solid line is the ground truth, the pink dashed line is the TF prediction result, and the green dashed line is the C-TF prediction result.

## 6. Conclusions

In this paper, we propose a method for PTV’s trajectory prediction under urban scenarios using LIDAR, camera, and combined inertial navigation system. The method combines the modeling of PTV detection, tracking, and trajectory prediction as an organic entirety. This approach is proven to be robust and it satisfies the requirement of trajectory prediction in the real urban road in Wuhan, China. The accurate and robust modeling of PTV trajectory prediction using multi-sensor fusion consists of several procedures that all have a significant influence on the final prediction results. To obtain the robust trajectory of the moving PTV from EV, YOLOv5 and deepSORT are employed to detection and tracking under EV coordinate system using the camera. Next the relative position of the PTV and the EV is obtained by LIDAR-camera fusion. Then, the PTVs are unified under the EV vehicle coordinate system and converted to the world coordinate system using the combined inertial navigation system. The proposed history trajectory generation method proves to be effective in tracking PTVs. After obtaining PTV’s historical trajectory, an S-G filter is employed to smooth the vehicle trajectory. In the trajectory prediction component, unlike previous works, the PTV’s trajectory is predicted based on two transformer networks, namely TF and C-TF. For the TF model, only the X and Y axis velocities are used as inputs. For C-TF, the velocities of the X and Y axis are clustered, and then the IDs of the clusters are one-hot encoded for training in the world coordinate system. The aim of the method is to dig deep into the process of predicting PTV’s trajectory using LIDAR, camera, and combined inertial navigation system under real driving scenarios to find a better way to organize the entire information flow. The method is tested using the image, point cloud and longitude and latitude, and the parameters of the models are optimized using the experimental method. The results reveal that the proposed two transformer-based methods achieve higher prediction accuracy compared with the LSTM-based method. In addition, the ADE for C-TF and TF are 1.699 m and 2.708 m, respectively, which improve 10.460 m and 8.271 m, respectively, compared to LSTM. The FDE for C-TF and TF are 3.519 m and 6.046 m, respectively, which improve 20.781 m and 18.254 m respectively compared to LSTM. The proposed C-TF method has greater improvements in ADE, FDE and X-axis than TF model while TF method offers slightly better effects than C-TF in Y axis. In conclusion, both C-TF and TF have significant improvements compared to LSTM when using ADE and FDE as evaluation metrics, and C-TF yields more accurate trajectory predictions than TF. Future work will detect lane lines using the camera and using future trajectory and lane lines to identify PTV driving intentions.

## Figures and Tables

**Table 1:** The ADE and FDE of LSTM, TF, and C-TF on real-world preceding vehicles dataset.

	LSTM		TF		C-TF	
	ADE (m)	FDE (m)	ADE (m)	FDE (m)	ADE (m)	FDE (m)
1s	4.244	8.090	1.042	1.495	0.737	1.056
2s	8.596	16.960	1.816	3.642	1.204	2.244
3s	12.159	24.300	2.708	6.046	1.699	3.519

**Table 2:** The ADE and FDE of LSTM, TF, and C-TF on the Argoverse dataset.

	LSTM		TF		C-TF	
	ADE (m)	FDE (m)	ADE (m)	FDE (m)	ADE (m)	FDE (m)
1 s	2.76	5.52	0.72	0.88	0.60	0.67
2 s	5.86	11.73	1.00	1.68	0.79	1.35
3 s	8.32	16.59	1.30	2.49	1.11	2.38

## Data Availability

Not applicable.
